# Phases Hybriding and Hierarchical Structuring of Mesoporous TiO_2_ Nanowire Bundles for High‐Rate and High‐Capacity Lithium Batteries

**DOI:** 10.1002/advs.201500070

**Published:** 2015-05-08

**Authors:** Jun Jin, Shao‐Zhuan Huang, Jing Liu, Yu Li, Li‐Hua Chen, Yong Yu, Hong‐En Wang, Clare P. Grey, Bao‐Lian Su

**Affiliations:** ^1^ Laboratory of Living Materials at the State Key Laboratory of Advanced Technology for Materials Synthesis and ProcessingWuhan University of Technology122 Luoshi Road430070WuhanHubeiChina; ^2^ Department of ChemistryUniversity of CambridgeLensfeild RoadCambridgeCB2 1EWUK; ^3^ Laboratory of Inorganic Materials Chemistry (CMI)University of Namur61 rue de BruxellesB‐5000NamurBelgium

**Keywords:** hierarchical structuring, lithium‐ion batteries, mesoporous nanowire bundles, phases hybriding, TiO_2_

## Abstract

A hierarchical mesoporous TiO_2_ nanowire bundles (HM‐TiO_2_‐NB) superstructure with amorphous surface and straight nanochannels has been designed and synthesized through a templating method at a low temperature under acidic and wet conditions. The obtained HM‐TiO_2_‐NB superstructure demonstrates high reversible capacity, excellent cycling performance, and superior rate capability. Most importantly, a self‐improving phenomenon of Li^+^ insertion capability based on two simultaneous effects, the crystallization of amorphous TiO_2_ and the formation of Li_2_Ti_2_O_4_ crystalline dots on the surface of TiO_2_ nanowires, has been clearly revealed through ex situ transmission electron microcopy (TEM), high‐resolution transmission electron microscopy (HRTEM), X‐ray diffraction (XRD), Raman, and X‐ray photoelectron spectroscopy (XPS) techniques during the Li^+^ insertion process. When discharged for 100 cycles at 1 C, the HM‐TiO_2_‐NB exhibits a reversible capacity of 174 mA h g^−1^. Even when the current density is increased to 50 C, a very stable and extraordinarily high reversible capacity of 96 mA h g^−1^ can be delivered after 50 cycles. Compared to the previously reported results, both the lithium storage capacity and rate capability of our pure TiO_2_ material without any additives are among the highest values reported. The advanced electrochemical performance of these HM‐TiO_2_‐NB superstructures is the result of the synergistic effect of hybriding of amorphous and crystalline (anatase/rutile) phases and hierarchically structuring of TiO_2_ nanowire bundles. Our material could be a very promising anodic material for lithium‐ion batteries.

## 1 Introduction

Considerable attention has been paid on electrochemical energy storage devices, especially rechargeable lithium‐ion batteries (LIBs), with both high power and high energy densities because of the applications in electric vehicles and portable electronic devices.^1^, ^2^, ^3^, ^4^, ^5^ However, many potential electrode materials of LIBs are limited by slow Li‐ion diffusion, poor electron transport in electrodes, and increased resistance at the electrode/electrolyte interface at high discharge–charge rates.^1^, ^6^ Various research has investigated nanostructures (e.g., nanoscale size, nanoporous, or hierarchically nano/macrostructure) to improve the electrochemical performances by providing good access of electrolyte to the electrode surface, shortening the Li^+^ insertion/extraction pathway, and facilitating charge across the electrode/electrolyte interface, resulting in excellent capacity, long cycle life, and good rate performance.^7^, ^8^, ^9^, ^10^, ^11^, ^12^, ^13^

Recently, Ti‐based nanostructures (including TiO_2_, hydrogen trititanate, Li_4_Ti_5_O_12_, etc.) have received increasing attention as promising Li‐ion battery anode materials owing to their low cost, non‐toxicity, low volume change, excellent recharge ability, improved safety over graphite, and relatively high lithium insertion potential (1.5–1.8 V vs Li/Li^+^).^4^, ^12^, ^14^, ^15^, ^16^, ^17^, ^18^, ^19^, ^20^ Using ultrafine nanocrystalline anatase or rutile, the Li insertion host can efficiently enhance the lithium storage capacity even at high discharge–charge current densities due to the shortened diffusion length and increased number of active sites for Li^+^ insertion.^21^, ^22^ Particularly, porous TiO_2_ nanostructures have attracted special interest for LIBs due to their unique properties such as high specific surface area, narrow pore size distribution, and good permeation, which are helpful for the improvement of cycling stability and capacity at high charge–discharge rates.^21^, ^23^, ^24^ Most recently, we found that the presence of straight inner‐particle mesochannels in 3D ordered macro‐mesoporous (3DOMM) TiO_2_ offers continuous and shorter path lengths for Li ions diffusion compared to wormlike crystallite aggregated mesopores present in 3D ordered macroporous (3DOM) TiO_2_.^25^

In addition, the amorphous phase of TiO_2_ has recently aroused much attention for improving the capacities of the electrode materials^26^, ^27^, ^28^, ^29^ in spite of the fact that the amorphous phase may not be stable enough for the electrochemical reaction. Borghols et al.^28^ prepared amorphous TiO_2_ from the hydrolysis of titanium isopropoxide. The obtained amorphous TiO_2_ nanostructure has a high initial discharge capacity of 810 mA h g^−1^. However, only ≈25% of the capacity appears to be reversible after 50 cycles due to the unstable amorphous structure. Gao et al.^30^ reported the first direct chemical and imaging evidence of lithium‐induced atomic ordering in the amorphous TiO_2_ anode. They found a new reaction mechanism on the formation of cubic Li_2_Ti_2_O_4_ for the lithiation behavior of the amorphous nanotube during the lithiation process. These results suggest that a material‐containing amorphous phase on the crystalline TiO_2_ nanostructures should be a good candidate for the enhancement of capacity and stability for LIBs. However, in Gao's work, they did not mention the function of cubic Li_2_Ti_2_O_4_ for the performance of LIBs.^30^ Thus, it will be very interesting to understand the contribution of the formed cubic Li_2_Ti_2_O_4_ during the discharge–charge process for LIBs.

All the above studies suggest that a hierarchically porous crystalline TiO_2_ nanostructure with an amorphous surface and straight channels in nanoscale should be a promising material for high performance of lithium storage and cycling stability. The amorphous phase can improve the specific surface area and provide more active sites for Li^+^ insertion, leading to the high Li^+^ storage capability. The straight nanochannels can facilitate the Li^+^ diffusion and the Li^+^ insertion/extraction process. Furthermore, the crystalline phases inside the hierarchical structure supply a stable host for the Li^+^ insertion/extraction reaction and facilitate charge transfer at the electrode/electrolyte interface, resulting in long cycle life and high rate performances.

Herein, we demonstrate the design and synthesis of hierarchically structured mesoporous TiO_2_ anatase/rutile hybrid nanowire bundles (HM‐TiO_2_‐NB) with amorphous phase at the nanowire outlayer surface and uniform straight nanochannels at a low temperature under acidic and wet conditions using triblock copolymer P123 as a mesoporous template. Such super hierarchical structures used as anode materials in lithium‐ion batteries exhibit high reversible capacity, excellent cycling performance, and superior rate capability. More interestingly, a self‐improving phenomenon of the Li^+^ insertion capability based on the crystallization of amorphous TiO_2_ and the formation of new Li_2_Ti_2_O_4_ crystal islands in the HM‐TiO_2_‐NB super structure has been revealed during the discharge–charge processes. The synergy of the amorphous/crystalline (anatase–rutile) phases hybriding and the hierarchical structuring of mesoporous TiO_2_ nanowire bundles leads to the large enhancement of the electrochemical performances and the self‐improving phenomenon.

## 2 Results and Discussion

**Figure 1:**
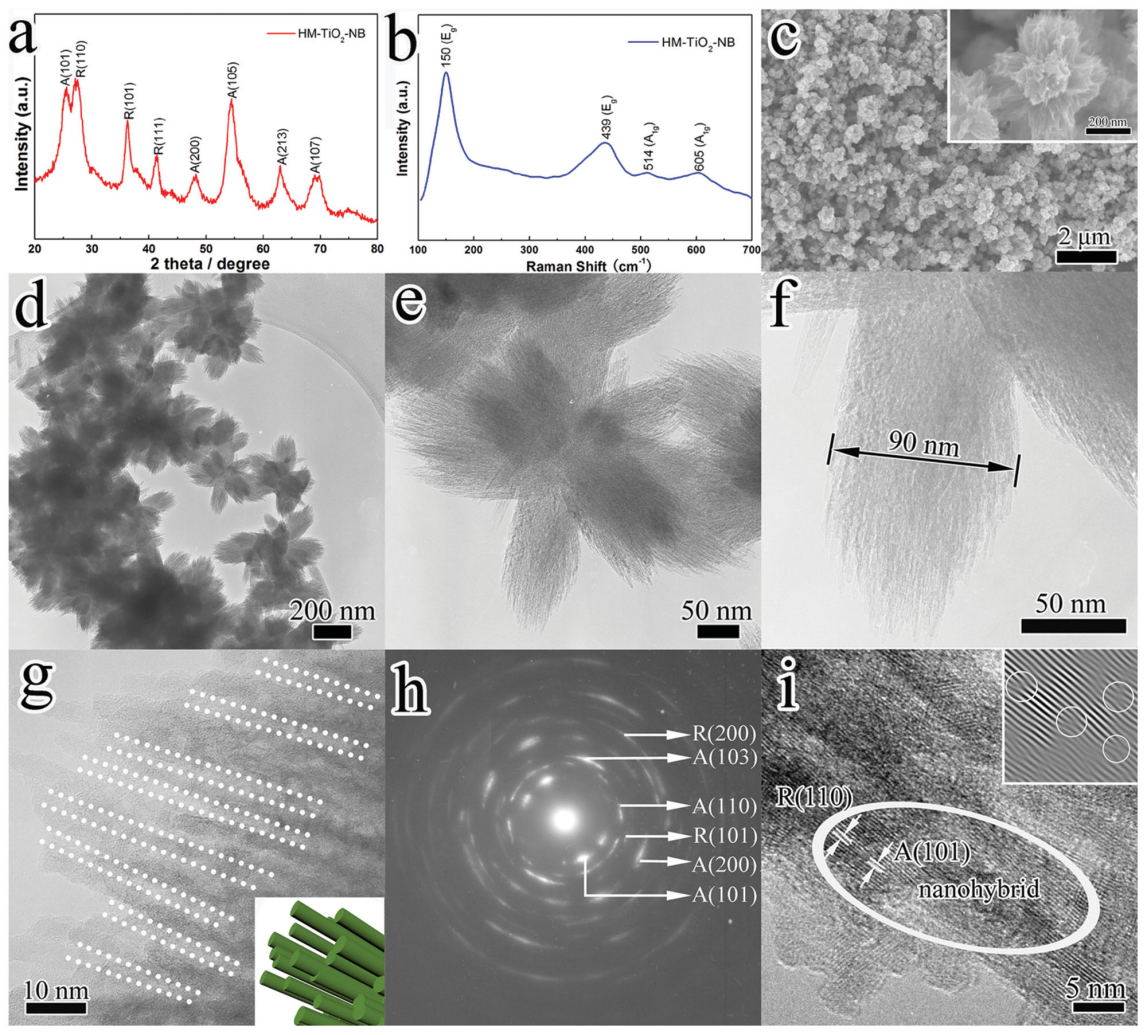
The characterizations of the as‐prepared HM‐TiO_2_‐NB sample. a) XRD patterns; b) Raman spectrum; c) SEM image; d–f) TEM images; g) HTREM image showing nanochannels; h) the corresponding SAED of (g) and i) HRTEM image. The inset in (c) is the close‐view SEM image. The inset in (g) is the schemed tip of one bundle. The inset in (i) is the reverse process image coming from the FFT pattern of the “nanohybrid” region.

**Figure** **1**a shows the X‐ray diffraction (XRD) patterns of the as‐prepared HM‐TiO_2_‐NB sample. The diffraction peaks at 25.3°, 37.9°, 48.1°, 54.1°, 62.8°, and 75.3° can be indexed to the (101), (103), (200), (105), (213), and (107) planes of anatase (JCPDS card no. 21‐1272, space group *I*4_1_/amd), respectively. The other peaks at 27.3°, 36.1°, and 41.3° can be attributed to the (110), (101), and (111) planes of rutile (JCPDS card no. 84‐1284, space group P42/mnm), respectively. The XRD patterns indicate that the obtained HM‐TiO_2_‐NB structure is a mixture of anatase and rutile phases. In addition, all the reflections have a large half‐peak width and the XRD pattern is slightly rough, indicating the low crystallinity with the possible existence of the amorphous region or the small crystallites in the HM‐TiO_2_‐NB nanowire‐bundled structure. It can be calculated that the weight ratio of anatase and rutile is ≈43:57.^31^ The coexistence of the anatase and rutile TiO_2_ phases in the HM‐TiO_2_‐NB structure was further proven by the Raman spectroscopic analysis (Figure 1b). Due to the different crystal structures and space groups, the anatase and rutile phases demonstrate different characteristic spectra. The peaks at 150 (*E*_g_) and 514 (*A*_1g_) cm^−1^ are attributed to the anatase phase,^32^, ^33^ while the other peaks at 439 (*E*_g_) and 605 (*A*_1g_) cm^−1^ can be indexed to the rutile phase, respectively.^33^, ^34^

Figure 1c shows the scanning electron microscopy (SEM) images of the as‐prepared HM‐TiO_2_‐NB sample, which is composed of numerous well‐defined flower‐like superstructures of about 500–600 nm in diameter, which are constructed by the hierarchical assembly of several nanowire bundles of a diameter of 50–100 nm (Figure 1c, inset). Each bundled structure consists of many 1D nanowires, which self‐assemble into bundles leading to the formation of porous straight nanochannels, along with straight nanowires (Figure S1, Supporting Information).

Transmission electron microcopy (TEM) observations were carried out to present more detailed information of the as‐prepared HM‐TiO_2_‐NB sample. Figure 1d–i clearly shows that the HM‐TiO_2_‐NB superstructure is composed of numerous nanowire bundles. Well‐defined straight nanochannels of 4–6 nm among and along with nanowires of 3–5 nm in diameter are clearly visible (Figure 1e–g). The bundles in the HM‐TiO_2_‐NB sample are radially oriented from the central region toward the edges of the HM‐TiO_2_‐NB structure. The selected area electron diffraction (SAED, Figure 1h) patterns from one bundle of TiO_2_ nanowires reveal a set of spots corresponding to (101), (110), (103), and (200) planes of the anatase phase and (101) and (200) planes of the rutile phase, respectively, being in agreement with the XRD data and Raman spectroscopic study (Figure 1a,b). The dispersion pattern from the central spot indicates that there is amorphous phase in the TiO_2_ nanowire bundles. A close view of the bundle of Figure 1f displays that each crystalline anatase/rutile TiO_2_ nanowire is enveloped by an amorphous TiO_2_ layer (Figure S2, Supporting Information). Figure 1i shows the high‐resolution transmission electron microscopy (HRTEM) image of the bundles, which demonstrates that the distances of the lattice spacing measured to be 0.32 and 0.35 nm, corresponding to the (110) plane of rutile phase and the (101) plane of anatase phase, respectively. It is interesting to note that both anatase and rutile phases appear in the same nanowire. After careful observations, one can see that there are many edge dislocations between the rutile phase and the anatase phase, which results from the two phases being present simultaneously in the same nanowire. To better show the edge dislocation between the two phases, the reverse process image coming from the fast Fourier transform (FFT) pattern of the “nanohybrid” region was performed, which clearly displays the edge dislocations in the nanowires (Figure 1i, inset), indicating many alternated rutile and anatase nanodomains in the single crystalline nanowires. In fact, the co‐existence of the crystalline (anatase and rutile) and amorphous phases in the same nanowire is commonly observed in the as‐prepared HM‐TiO_2_‐NB sample in our intensive observations (Figure S3, Supporting Information). Such a superstructure with hybrid nanowires can provide, on one hand, higher specific surface areas and more active sites for the Li^+^ insertion/extraction and promote the Li^+^ storage properties. On the other hand, each core of nanowire is formed by crystalline structures (mixture of anatase and rutile) of TiO_2_, which are a stable host for the Li^+^ insertion/extraction reaction and facilitate charge across the electrode/electrolyte interface.

**Figure 2:**
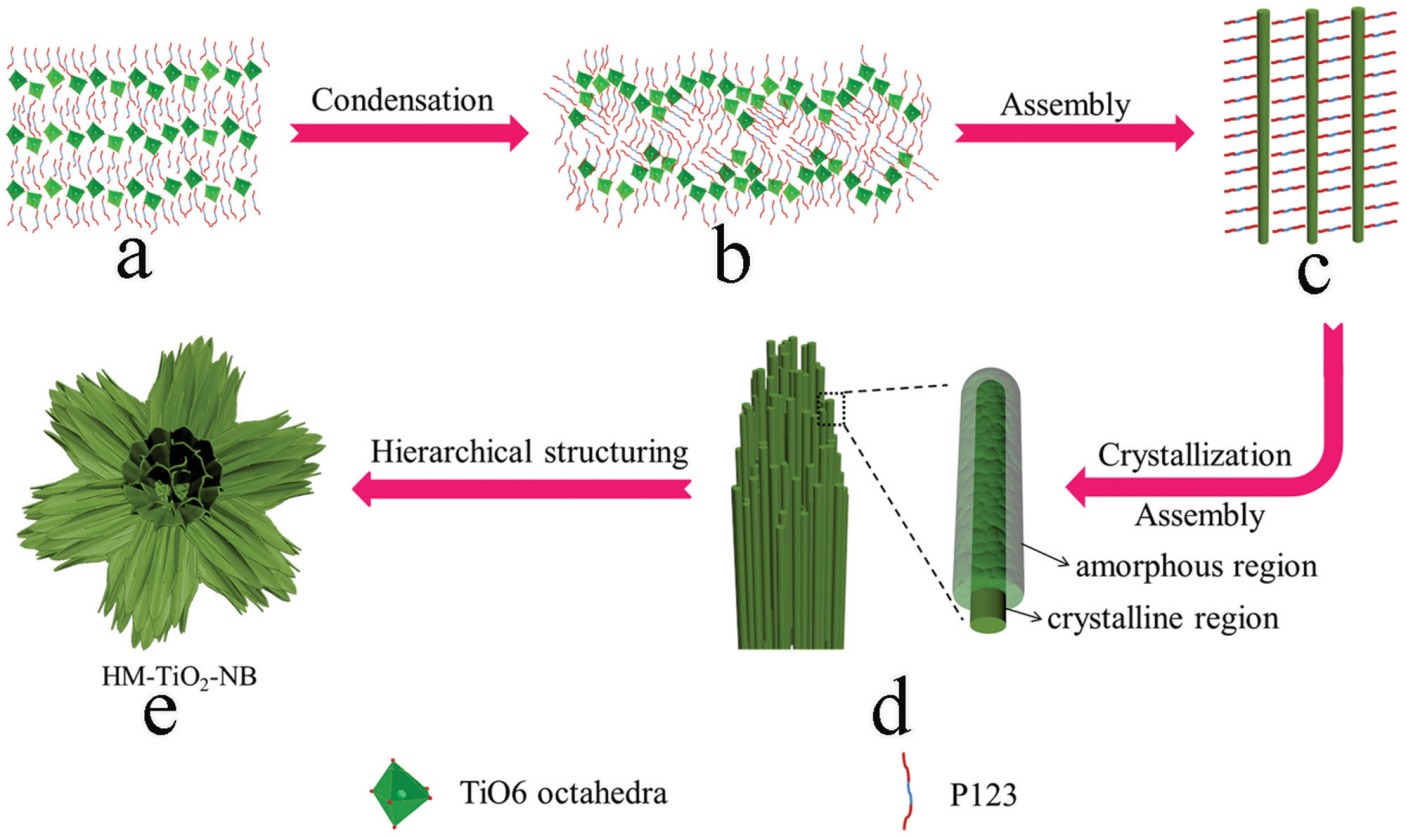
Schematic illustration of the synthesis mechanism of the hierarchical structures of the as‐prepared HM‐TiO_2_‐NB structure. a) the self‐assemble of the hydrolyzed titanium precursor molecules with the triblock copolymer P123 surfactant molecules to form a lamellar mesophase; b) the formation of hybrid system surfactant‐TiO2 nanoparticles; c) the formation of a 3D superstructure of surfactant nanowires; d) and e) the hierarchical structuration of bundles of mesoporous amorphous/crystalline TiO_2_ nanowires.

Our results demonstrate that the presence of concentrated hydrochloric acid is essential for the formation of anatase and rutile phases at low reaction temperature (80 °C). Since without the addition of hydrochloric acid, only amorphous nanoparticles with different sizes appear in the final product (Figure S4, Supporting Information). Under our reaction system, the copoly­mer P123 and humidity can control the hydrolysis and condensation of the titanium isopropoxide precursor.^35^, ^36^ Based on the observations mentioned above, a possible formation mechanism of the HM‐TiO_2_‐NB superstructure has been illustrated in **Figure** **2**. At the very beginning of the reaction, the hydrolyzed titanium precursor molecules bind with the triblock copoly­mer P123 surfactant molecules, which self‐assemble to form a lamellar mesophase in order to reduce the surface energy (Figure 2a). With the volatilization of ethanol and the sluggish hydrolysis of the titanium isopropoxide precursor, the condensation of hydrolyzed TiO_2_ precursor occurs accompanied by the decrease of the charge density of TiO_2_ at the interface. This process is caused by the increase of the interfacial curvature of the surfactant matrix,^37^ leading to the formation of hybrid system surfactant‐TiO_2_ nanoparticles (Figure 2b). The further condensation of aligned nanoparticles leads to a 3D superstructure of surfactant nanowires (Figure 2c). Meanwhile, both anatase and rutile phases from the core of nanowires are formed in the presence of concentrated hydrochloric acid at 80 °C to give nanodomains of anatase and rutile phases.^35^, ^36^, ^38^, ^39^ However, due to the low reaction temperature, the amorphous TiO_2_ cannot be totally converted to the crystalline phase giving rise to an amorphous layer at the outer surface of each nanowire.^35^, ^39^, ^40^, ^41^ Finally, a superstructure based on the hierarchical structuration of bundles of mesoporous amorphous/crystalline TiO_2_ nanowires was obtained (Figure 2d,e). The self‐assembly of numerous straight nanowires into a bundle produces homogeneously sized straight nanochannels among and along with straight nanowires (Figures 1g, 2d), as confirmed by the N_2_ adsorption/desorption isotherms shown in Figure S5a (Supporting Information). It gives a type‐IV isotherm with a type H3 hysteresis loop, indicating a mesoporous structure.^42^ The HM‐TiO_2_‐NB has a high specific BET surface area of 117.8 m^2^ g^−1^ with a pore size centered at 6 nm (Figure S5b, Supporting Information), being in well consistent with the TEM observation (Figure 1h).

Generally, the principle reaction that governs the electrochemical processes in nanostructured TiO_2_/Li half‐cell is thought to be

(1) $$x{\mathrm{Li}}^{+}+xe^{-}+{\mathrm{TiO}}_{2}↔{\mathrm{Li}}_{x}{\mathrm{TiO}}_{2}$$

corresponding to the complete reduction of Ti^4+^ to Ti^3+^ and a theoretical capacity of anatase TiO_2_ of 335 mA h g^−1^.^43^ Upon lithium insertion, the elastic interaction force between intercalated lithium ions and the formation of weak Ti–Ti interactions results in a phase transition from the original tetragonal phase to the Li_0.5_TiO_2_ phase with orthohombic symmetry. The lithium ions are randomly distributed over half of the available interstitial octahedral sites. Therefore, from a practical point of view, the maximum reversible insertion level is limited to an insertion coefficient of 0.5 Li per formula unit of TiO_2_, corresponding to the two phases equilibrium between the Li‐poor Li_0.05_TiO_2_ and the Li‐rich Li_0.5_TiO_2_ phases. The associated reversible capacity is generally 167 mA h g^−1^.^44^, ^45^, ^46^, ^47^, ^48^, ^49^ The rutile phase is the stable form of TiO_2_. It has been observed that microcrystalline rutile TiO_2_ does not show an excellent Li storage property, due to the peculiar tetragonal structure, which does not facilitate Li^+^ transport along the *ab*‐plane in comparison to that along the *c*‐axis.^9^, ^24^, ^50^ However, nano‐sized rutile TiO_2_ with various morphologies demonstrated an insertion coefficient of ≈0.5 mol of Li per TiO_2_,^51^ which is similar to the practical capacity of anatase phase. Recently, it has been reported that nanostructured rutile can strongly enhance both the capacity and the rate capability,^14^, ^26^ indicating that the nanosized rutile structures could be the promising anode materials. Moreover, it has been shown that the presence of high surface amorphous TiO_2_ can enhance the Li ions storage capacities owing to a pseudocapacitance effect.^25^, ^26^, ^27^, ^28^ Finally, the presence of straight nanochannels is also very favorable for Li ions diffusion.^30^ Therefore, our material with the large number of nanowires containing a mixture of anatase and rutile nanodomains covered by high surface area amorphous phase and the straight nanochannels can supply a large contact surface between the electrolyte and anode material, important active sites for Li storage and shorten the Li^+^ diffusion paths and the electron transport lengths.^9^, ^16^, ^52^

**Figure 3:**
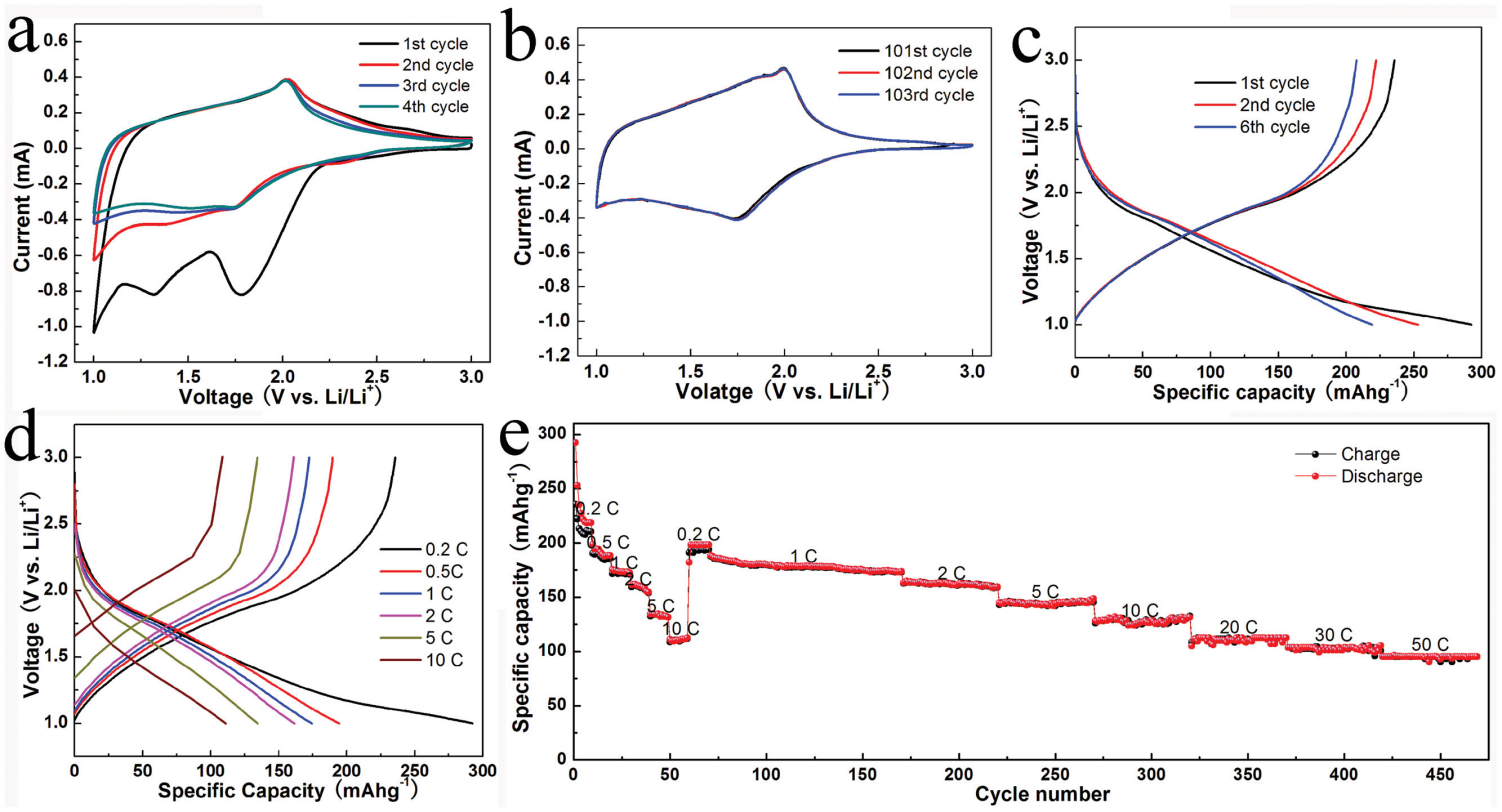
The electrochemical properties of the as‐prepared HM‐TiO_2_‐NB sample. a) Representative CVs at a scan rate of 0.5 mV s^−1^ for the first four cycles; b) representative CVs at a scan rate of 0.5 mV s^−1^ for the 101st, 102nd, and 103rd cycles; c) discharge–charge profiles at a current rate of 0.2 C for the first, second, and sixth cycles; d) discharge–charge profiles at various current rates for the first cycle; e) rate and cycle performances at different rates.

**Figure** **3**a shows the representative cyclic voltammograms (CVs) of the HM‐TiO_2_‐NB electrode at 0.5 mV s^−1^ for the first few cycles at 1–3 V (vs Li/Li^+^), displaying redox reaction at 1.3 V and 1.8 V (vs Li/Li^+^) during discharge and at 2.0 V during charge. The peaks at 1.75 V (cathodic sweep) and 1.9 V (anodic sweep) correspond to the Ti^4+^/Ti^3+^ redox reaction in the anatase phase^9^, ^11^ that exists in the HM‐TiO_2_‐NB (Figure 1a). The peak at 1.3 V (cathodic sweep) is observed in the first cycle and weakened rapidly during the following cycles, which can be attributed to the irreversible formation of LiTiO_2_ phase from rutile phase.^16^, ^25^ With the increase of charge–discharge cycles, the intensities of the peaks at 1.3 V and 1.8 V (cathodic sweep) decrease, while the intensities of peaks at 2.0 V (anodic sweep) remain unchanged. Furthermore, when the electrode is charged for more than 100 cycles, the peaks at 1.8 V (cathodic sweep) and 2.0 V (anodic sweep) remain unchanged (Figure 3b), indicating the high stability structure of the HM‐TiO_2_‐NB.

Figure 3c depicts the discharge–charge voltage profiles of the HM‐TiO_2_‐NB electrode for the first few cycles at a current of 0.2 C (1 C = 167 mA g^−1^). The results show that the HM‐TiO_2_‐NB can accommodate Li up to Li_0.88_TiO_2_ (293 mA h g^−1^) during the first discharge process. Subsequently, 0.7 mol of Li per 1 mol TiO_2_ (Li_0.7_TiO_2_, 236 mA h g^−1^) can be cycled reversibly, leading to a high Coulombic efficiency of 81%. The discharge curve in the first cycle can be divided into two stages. In the first stage, it is observed that the voltage decreases monotonically from the open circuit sites of ≈2.9 V to ≈1.8 V, corresponding to a Li^+^ insertion capability of 34 mA h g^−1^. In this region, lithium is stored at the surface or inserted into the amorphous surface layer. In the second stage, it can be observed that the potential decreases slowly from 1.8 V to 1.0 V, leading to a Li^+^ capability of 259 mA h g^−1^. The lithium capacity in the second stage corresponds to lithium insertion into the TiO_2_ lattice and further insertion of Li^+^ into the surface layer of the electrode material. Remarkably, there is only a very short plateau (not obvious) compared with that previously reported in the literature.^16^ The sloped behavior in the second region is in good agreement with the aforementioned CV results and is more likely to that of the nano‐sized rutile^24^, ^25^, ^53^ and amorphous TiO_2_ 26. As mentioned above, anatase and rutile phases exist and alternatively appear in the same nanowire frequently, and amorphous is at the surface of the nanowires (Figures 1, S3, Supporting Information). Thus, the formed amorphous/crystalline hybrid nanowires are normally the reason for the very short plateau and the long sloped behavior of discharge processes.^9^, ^26^, ^27^ Due to this special superstructure, it was highly reversible during the following discharge–charge cycles, leading to an excellent capacity retention rate. In the second cycle, the discharge capacity decreased to 253 mA h g^−1^ with a corresponding charge capacity of 222 mA h g^−1^, leading to a Coulombic efficiency of 88%. In the sixth discharge–charge cycle, the Coulombic efficiency even increased to 95%.

Figure 3d displays the discharge–charge voltage profiles of the HM‐TiO_2_‐NB electrode at various current densities. The discharge‐specific capacities of 292, 199, 175, 162, 134, and 112 mA h g^−1^ are obtained when the current rates are 0.2, 0.5, 1, 2, 5, and 10 C, respectively. Figure 3e shows the rate capabilities of the HM‐TiO_2_‐NB electrode at various current densities. It is important to note that an initial discharge capacity of 292 mA h g^−1^ could be obtained at a current rate of 0.2 C, leading to a lithium insertion coefficient of 0.88, which is much higher than the theoretical value of 0.5.^25^, ^53^ This high initial discharge capacity can generally be attributed to surface phenomena, such as the pseudocapacitance effect in the porous amorphous phase. The synergy of the amorphous phase, high surface area, and straight porous nanochannels permit the HM‐TiO_2_‐NB electrode to have a discharge capacity of 112 mA h g^−1^ even at a rate of 10 C. Although the sluggish kinetic of additional surface capacity, with its increase in current rate due to the tardiness of lithium storage at the electrode surface, decreases the total storage capacity of the electrode, the extraordinarily high capacity observed at the rate of 10 C should be assigned to the very high specific surface area and the amorphous phase of our HM‐TiO_2_‐NB superstructure providing more active sites for lithium insertion. The straight nanochannels in each nanowire bundle can provide shorter lengths for Li^+^ diffusion and electron transport, as well as quick liquid electrolyte penetration. A capacity of 199 mA h g^−1^ was resumed while the current density was reduced back to 0.2 C again, indicating the very high stability of the HM‐TiO_2_‐NB electrode. Thus, the second round cycle performance was performed on the same LIB cell. Obviously, the HM‐TiO_2_‐NB superstructure demonstrates excellent capacity retention at various current densities. When charged at 1 C, the HM‐TiO_2_‐NB has an initial discharge capacity of 189 mA h g^−1^ and a subsequent charge capacity of 188 mA h g^−1^, leading to an irreversible capacity loss as low as 0.4%. Very interestingly, a self‐improving phenomenon of the Li^+^ insertion capability of the HM‐TiO_2_‐NB material during the ultra‐long discharge–charge cycles was observed. The initial discharge capacity of 189 mA h g^−1^ in the cycling region is higher than that of 175 mA h g^−1^ in the rating region at 1 C. Figure S6 (Supporting Information) shows the discharge–charge profiles of HM‐TiO_2_‐NB anode material at 1 C in the rating and cycling processes, respectively. When discharged in the rating process, the HM‐TiO_2_‐NB anode material demonstrates capacities of 34 mA h g^−1^ for the first stage and 141 mA h g^−1^ for the second stage, respectively. However, when discharged at 1 C in the cycling process, the HM‐TiO_2_‐NB anode material possesses capacities of 34 mA h g^−1^ for the first stage and 155 mA h g^−1^ for the second stage, respectively. It clearly displays that the capacities at the second stage in the cycling process is higher than that in the rating process. According to previous research, this self‐improving phenomenon may be generated by the crystallization of amorphous TiO_2_ 54, 55 and/or the formation of Li_2_Ti_2_O_4_ crystal particles.^29^ When the HM‐TiO_2_‐NB electrode was discharged after 100 cycles, a very high reversible capacity of 174 mA h g^−1^ was retained, indicating the excellent electrochemical and structural stabilities of the HM‐TiO_2_‐NB anode material. This corresponds to a lithium coefficient of 0.52, which is slightly higher than the previously determined maximum value of 0.5. Its reversible volumetric capacity at 1 C after 100 cycles is ≈283.6 mA h cm^−3^, according to the measured tap density value of 1.63 g cm^−3^. Figure S7 (Supporting Information) demonstrates the Coulombic efficiency of HM‐TiO_2_‐NB anode material at 1 C for 100 cycles. It clearly shows that the Coulombic efficiency almost retains at ≈100% for 100 cycles. The discharge capacity decreased to 163 mA h g^−1^ when the current density was increased to 2 C. When the electrode was charged/discharged at 2 C after 50 cycles, a reversible discharge capacity of 159 mA h g^−1^ was retained. The cycle performance shows that while charged at high current rates of 5, 10, 20, and 30 C after 50 cycles, the HM‐TiO_2_‐NB electrode is still able to deliver reversible capacities of 147, 131, 112, and 104 mA h g^−1^, respectively, which are much higher than many other TiO_2_ structures.^9^, ^12^, ^15^, ^16^ Remarkably, when the current density was increased to a very high value of 50 C, a superior reversible capacity of 96 mA h g^−1^ could still be delivered after 50 cycles. These extraordinarily high capacities and rate capabilities are among the best performances of TiO_2_ in the literature. These extraordinary performances can be ascribed to particular hierarchically structured mesoporous TiO_2_ amorphous/crystalline hybrid nanowire bundles. This special superstructure can provide enough spaces for the conductive phase to Li ions storage and shorten the Li^+^ diffusion paths and the electron transport lengths. Furthermore, the porous straight channel structure of the HM‐TiO_2_‐NB can further facilitate the electrolyte penetration and the lithium diffusion, as well as the electron transportation in the electrode.^25^, ^26^, ^28^, ^56^, ^57^ The high stability of electrochemical performances can be attributed to the crystalline regions inside the bundles of the HM‐TiO_2_‐NB sample. Thus, the amorphous, crystalline phases, and the hierarchical mesoporous nanowire bundled structure can synergistically enhance the Li^+^ insertion capability, high rate capacity, and cycle life.

**Figure 4:**
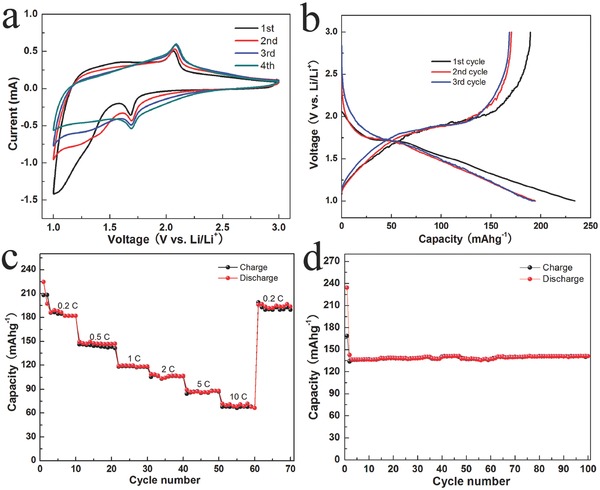
The electrochemical properties of the HM‐TiO_2_‐NB‐350 sample. a) Representative CVs at a scan rate of 0.5 mV s^−1^ for the first three cycles; b) discharge–charge profiles at a current of 0.2 C for the first, second, and sixth cycles; c) cycle performance at a current of 1 C; d) rate performance at various current densities.

To better understand the excellent electrochemical performance of HM‐TiO_2_‐NB material as an anode, the as‐prepared HM‐TiO_2_‐NB was heat treated at 350 °C (HM‐TiO_2_‐NB‐350) in the air. From the XRD (Figure S8a, Supporting Information), SEM (Figure S8b,c, Supporting Information), and TEM images (Figure S8d–f, Supporting Information), it can be observed that the hierarchical structure morphology can be generally retained, while the amorphous phase is completely transformed into crystalline phases accompanied by the broadening of the diameter of nanowires to 15 nm, leading to the decrease in number of straight nanochannels. Thus, the HM‐TiO_2_‐NB‐350 sample has a lower surface area of 72.4 m^2^ g^−1^ with a narrower pore size of 4–5 nm compared to the as‐prepared HM‐TiO_2_‐NB sample (Figure S5b, Supporting Information). The N_2_ adsorption–desorption isotherms are still of type IV with type H3 hysteresis (Figure S5a, Supporting Information). In the CV curves (**Figure** **4**a), the HM‐TiO_2_‐NB‐350 electrode has sharper peaks at 1.7 V (cathodic sweep) and 2.1 V (anodic sweep) than the HM‐TiO_2_‐NB electrode, indicating that the HM‐TiO_2_‐NB‐350 electrode has more obvious and stable redox potentials^58^, ^59^ due to the crystallization of the amorphous regions and the anatase‐to‐rutile phase during the heat treatment at 350 °C. In this case, two obvious voltage plateaus at ≈1.7 and ≈1.9 V can be observed during the discharge and charge processes of the HM‐TiO_2_‐NB‐350 electrode (Figure 4b), which are quite different from the HM‐TiO_2_‐NB electrode (Figure 3a). When the HM‐TiO_2_‐NB‐350 electrode was charged at 1 C (Figure 4c), a reversible capacity of 141 mA h g^−1^ was retained after 100 cycles. In Figure 4d, the HM‐TiO_2_‐NB‐350 electrode has discharge capacities of 225, 149, 120, 109, 89, and 72 mA h g^−1^ at rates of 0.2, 0.5, 1, 2, 5, and 10 C, respectively. Then, a capacity of 196 mA h g^−1^ was resumed when the rate was decreased back to 0.2 C. The comparison of the results of the HM‐TiO_2_‐NB‐350 sample with those of as‐prepared HM‐TiO_2_‐NB indicates clearly that the co‐existence of the amorphous and crystalline (anatase/rutile) phases in each nanowire, the very high surface area of HM‐TiO_2_‐NB superstructure, and the presence of a large number of straight nanochannels giving an easy accessibility of electrolyte and Li ions to nanowires are critical for advanced lithium storage.

**Figure 5:**
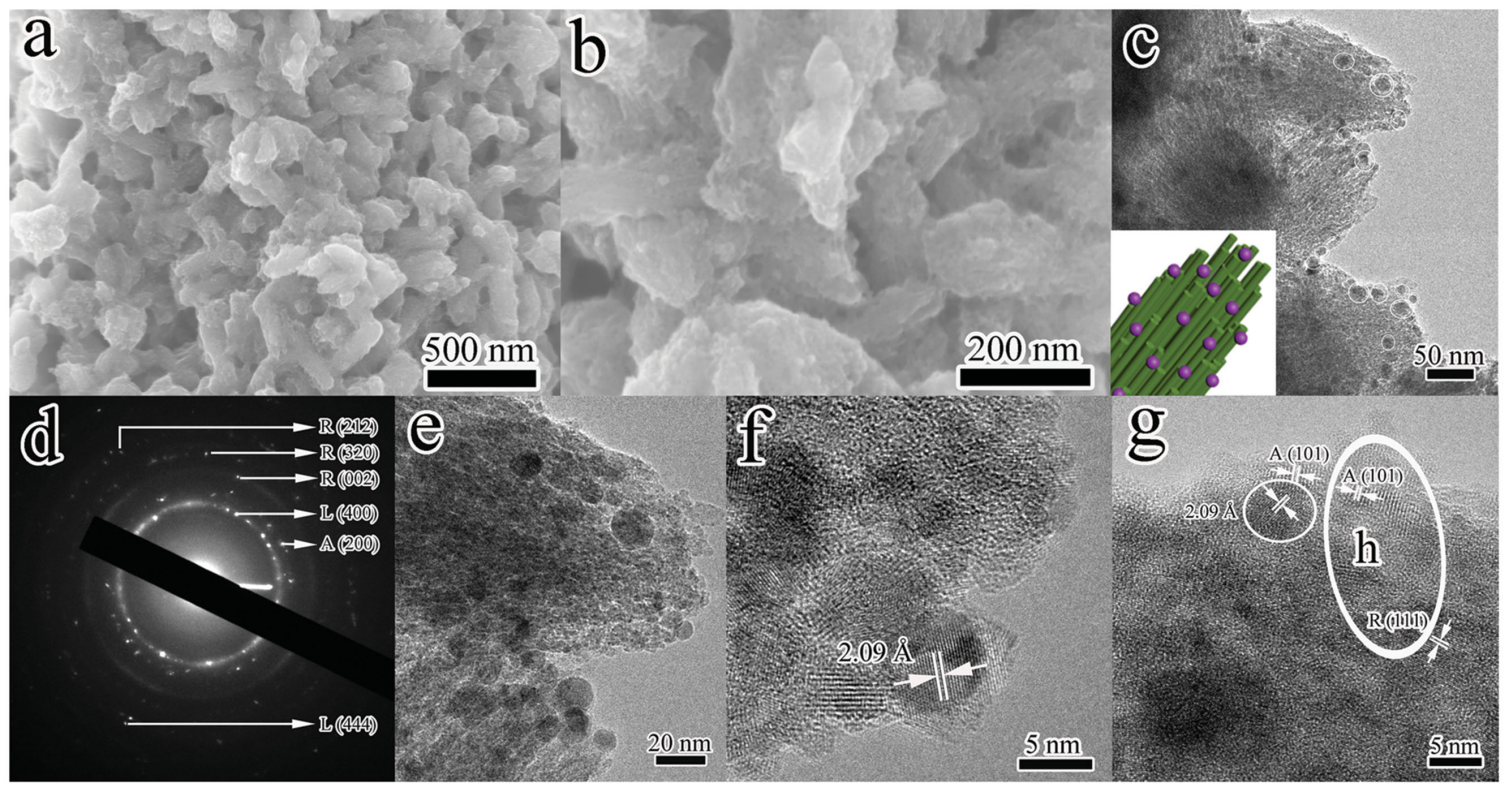
The SEM and TEM images of the HM‐TiO_2_‐NB electrode after 100 discharge–charge cycles at 1 C. a,b) SEM images; c) TEM image; d) the corresponding SAED; e–g) HRTEM images.

In order to further understand the advanced lithium storage property, the structure stability of the HM‐TiO_2_‐NB electrode, and the self‐improvement phenomenon of Li^+^ insertion capability, the post‐mortem studies were carried out after 100 discharge–charge cycles at 1 C rate. As shown in **Figure** **5**, the special flower‐like superstructure formed by the hierarchization of mesoporous TiO_2_ nanowire bundles is well retained (Figure 5a,b). A large number of straight nanochannels among nanowires are clearly visible (Figure 5c, inset). Interestingly, after the lithium insertion, small dots with a diameter of ≈5–10 nm can clearly be observed (Figure 5c). These dots are randomly distributed at the surface of the HM‐TiO_2_‐NB structure (Figure 5c,e). Figure 5d shows the corresponding SAED pattern of the HM‐TiO_2_‐NB anode material after 100 cycles. The electron diffraction rings in Figure 5d are indexed to anatase TiO_2_, rutile TiO_2_, and a new phase corresponding to Li_2_Ti_2_O_4_ (space group: *Fm3m*, lattice constants: *a* = *b* = *c* = 8.375 Å), respectively. HRTEM observation was performed on the lithiated HM‐TiO_2_‐NB sample. The lattice spacing of the particle in Figure 5f,g is measured to be 2.09 Å, corresponding to be the (400) plane of Li_2_Ti_2_O_4_, confirming the formation of crystalline Li_2_Ti_2_O_4_ phase.^29^, ^60^, ^61^ Therefore, Reaction (1) can be modified to contain the new crystalline phase of Li_2_Ti_2_O_4_ according to

(2) $$2(1-x){\mathrm{Li}}^{+}+2(1-x)e^{-}+2{\mathrm{Li}}_{x}{\mathrm{TiO}}_{2}↔{\mathrm{Li}}_{2}{\mathrm{Ti}}_{2}O_{4}$$

Based on the above TEM, HRTEM, and SAED results shown in Figure 5, it can be envisioned that the continuous lithium insertion of Li_x_TiO_2_ will lead to an atomic rearrangement in the HM‐TiO_2_‐NB structures (especially in the amorphous region) to form the new cubic crystals of Li_2_Ti_2_O_4_ corresponding to 1 Li inserted per formula unit of TiO_2_.^29^ Hence, this newly obtained Li_2_Ti_2_O_4_ crystal can further facilitate the Li^+^ insertion capability of the HM‐TiO_2_‐NB anode material during the electrochemical reaction, leading to the self‐improvement of the Li^+^ insertion capability at the same current density (e.g., 1 C, 2 C, even 10 C) during the cycling stage (after the rating stage), as shown in Figure 3e. After charged/discharged for more than 70 cycles (rating stage), the HM‐TiO_2_‐NB sample has a higher capacity of 189 mA h g^−1^ at 1 C at the cycling process than that at the rating process (175 mA h g^−1^). Moreover, when the current density was increased to 50 C for 50 cycles, a high capacity of 96 mA h g^−1^ is still retained.

Moreover, the distances of lattice spacing were measured to be 0.35 and 0.22 nm (region h in Figure 5g), corresponding to the anatase (101) plane and the rutile (111) plane. In addition, some amorphous layer can still be observed (Figure 5g). Furthermore, the straight channels constructed by anatase–rutile nanowires are still retained; all these observations indicate the highly structural and the electrochemical stability of the HM‐TiO_2_‐NB electrode.

**Figure 6:**
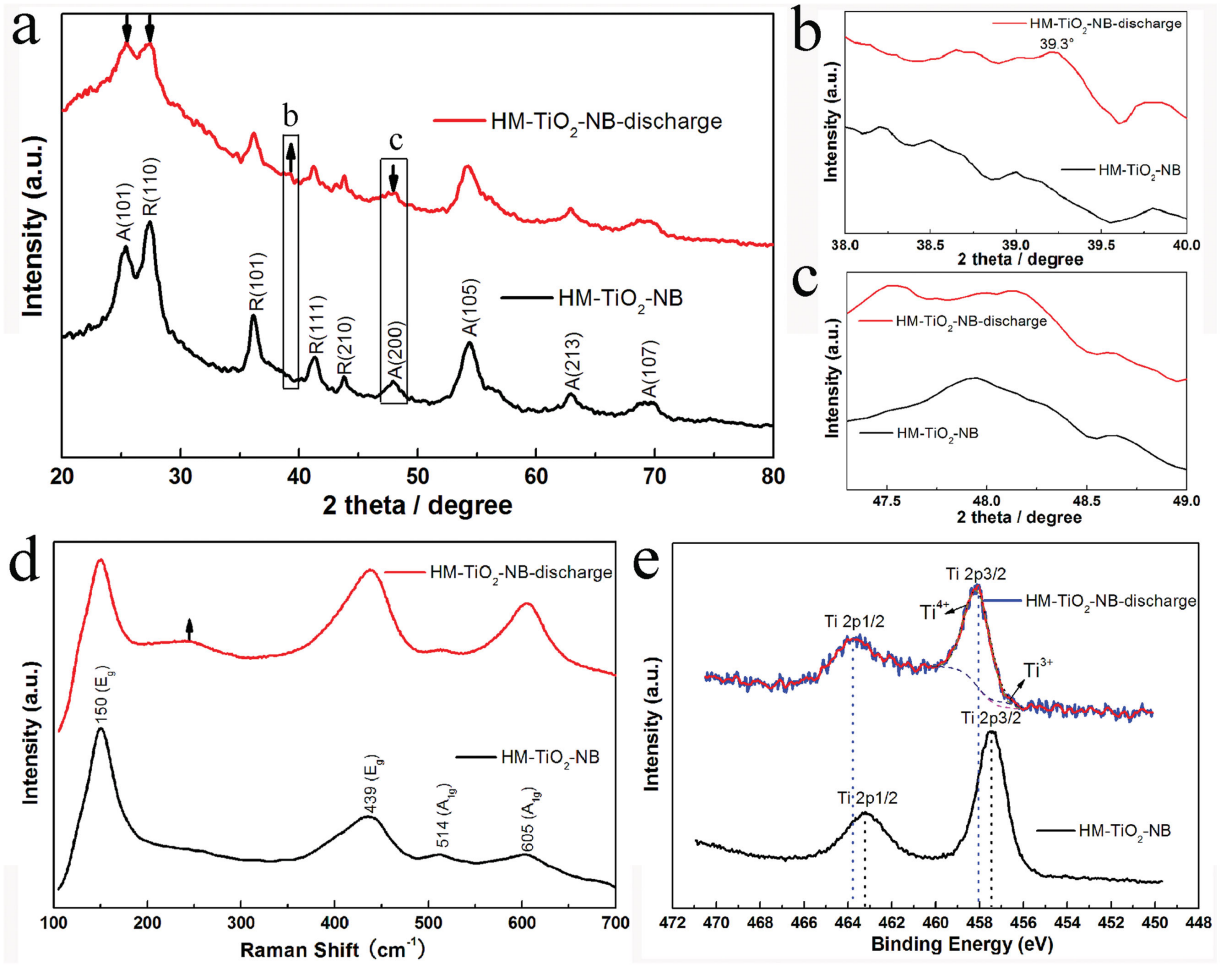
The ex situ XRD patterns, Raman spectra, and XPS spectra of the original HM‐TiO_2_‐NB and reacted HM‐TiO_2_‐NB anode material on the state of discharge. a) XRD patterns; b) the detailed XRD patterns of the highlighted region “b” in part (a); c) the detailed XRD patterns of the highlighted region “c” in part (a); d) Raman spectra; e) XPS spectra of the Ti 2p region.

**Figure 7:**
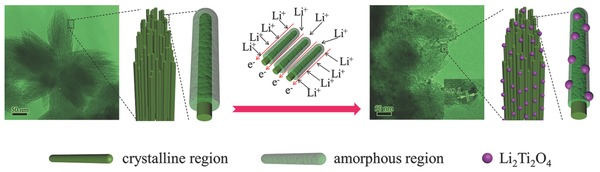
Schematic illustration of the Li^+^ insertion mechanism on the HM‐TiO_2_‐NB electrode and the formation of the Li_2_Ti_2_O_4_ crystalline phase.

Then, the ex situ XRD, Raman, and X‐ray photoelectron spectroscopy (XPS) techniques were utilized to detect the crystalline structure and the surface electronic state of the HM‐TiO_2_‐NB anode material on the state of discharge. The lithiated HM‐TiO_2_‐NB anode materials were recovered from the cycled half‐cell for ex situ XRD, Raman, and XPS characterizations. **Figure** **6**a–c shows the XRD patterns of the original HM‐TiO_2_‐NB material and the reacted HM‐TiO_2_‐NB anode material on the state of discharge (discharged to 1 V), respectively. Compared with the as‐prepared HM‐TiO_2_‐NB material, all the diffraction peaks of the reacted HM‐TiO_2_‐NB anode material from the initial anatase and rutile phases are weaker, especially the (101) plane of anatase and the (110) plane of rutile, indicating the Li^+^ insertion during the discharge process. Moreover, it can be observed that the peak of the anatase (101) plane decreases in intensity more rapidly than the peak of the rutile (110) plane. The appearance of diffraction peaks at 39.3° and 43.2° (Figure 6b) and the broadness of diffraction peaks at 48.0° (Figure 6c) indicate the formation of a Li*_x_*Ti_2_O_4_ titanate phase, which is well consistent with the previous research.^9^, ^29^, ^62^ Figure 6d shows the comparison of Raman spectra of the original HM‐TiO_2_‐NB material and the reacted HM‐TiO_2_‐NB anode material on the state of discharge, respectively. A new peak appears at ≈245.0 cm^−1^ in the reacted HM‐TiO_2_‐NB anode material, suggestive of the formation of the Li*_x_*Ti_2_O_4_ crystalline phases.^61^, ^63^, ^64^ Figure 6e shows the XPS spectra of the Ti 2p region of the surface of the original HM‐TiO_2_‐NB material and of the reacted HM‐TiO_2_‐NB anode material on the state of discharge, respectively. The binding energies of Ti 2p3/2 and 2p1/2 are observed at 457.5 eV and 463.2 eV, for as‐prepared HM‐TiO_2_‐NB material, corresponding to the Ti 2p3/2 and Ti 2p1/2 peaks of Ti^4+^, leading to a spin–orbit splitting of 5.7 eV.^65^, ^66^, ^67^ Then, for the reacted HM‐TiO_2_‐NB anode material on the state of discharge, these peaks shift to higher values of 458.1 eV and 463.9 eV, indicating the change of the surface electronic state of Ti element and the formation of the new Li*_x_*Ti_2_O_4_ crystalline phase. In particular, the HM‐TiO_2_‐NB on the state of discharge displays a typical Ti 2p3/2 core level XPS spectrum with Ti^4+^ (458.1 eV) and Ti^3+^ (456.7 eV) characteristics. This means that Li^+^ inserts into the “nanohybrid” regions of HM‐TiO_2_‐NB, leading to Li*_x_*TiO_2_ formation. The formed Li*_x_*TiO_2_ in the HM‐TiO_2_‐NB anode material has an enhanced electrical conductivity due to Ti^3+^ existence and further improves the Li^+^ storage performance.^68^, ^69^ On the basis of the post‐mortem characterization results mentioned above, a possible Li^+^ insertion mechanism into the hierarchical HM‐TiO_2_‐NB structure inducing the crystallization of amorphous phase and the formation of Li_2_Ti_2_O_4_ phase is proposed and illustrated in **Figure** **7**. The Li^+^ ions can quickly intercalate into the amorphous layer and nanodomains of nanowires in the HM‐TiO_2_‐NB material, leading to the formation of Li*_x_*TiO_2_ matrix and excellent Li^+^ insertion capability. Then, with the further Li^+^ insertion into the Li*_x_*TiO_2_ matrix, the disordered atoms of Li, Ti, and O rearrange into a new ordered Li_2_Ti_2_O_4_ crystalline structure to reduce the free energy of the system. This newly obtained Li_2_Ti_2_O_4_ crystal can further facilitate the Li^+^ insertion capability of the HM‐TiO_2_‐NB anode material during the discharge–charge process, leading to a self‐improving phenomenon of Li^+^ insertion capability.

## 3 Conclusions

By designing on the basis of hybriding of amorphous/crystalline phases in nanowires and hierarchical structuring of mesoporous bundled nanowires, a very promising anode material (HM‐TiO_2_‐NB) has been synthesized. This superstructure can provide more active sites for the Li^+^ insertion/extraction and facilitate the Li^+^ diffusion and the Li^+^ insertion/extraction process to promote the Li^+^ storage and electrochemical stability of the anode. The crystalline phases inside the hierarchical structure are a stable host and can facilitate charge across the electrode/electrolyte interface resulting in long cycle life and high rate performances. Compared with that presented in the literature, our material demonstrates superior electrochemical performances. The synergy of amorphous layer, mesoporous straight channels, and the crystalline nanodomains make a great enhancement of the electrochemical performances of TiO_2_. Through the nanowire amorphous surface, Li^+^ can continuously intercalate into the HM‐TiO_2_‐NB structure to form an amorphous Li*_x_*TiO_2_ matrix and a new Li_2_Ti_2_O_4_ crystal with a size range of 5–10 nm. This newly obtained Li_2_Ti_2_O_4_ crystal can further facilitate the Li^+^ insertion capability of the HM‐TiO_2_‐NB anode material during the discharge–charge process, leading to a self‐improving phenomenon of Li^+^ insertion capability. Our results indicate that HM‐TiO_2_‐NB with amorphous surface and straight channels is a good candidate for advanced lithium storage.

## 4 Experimental Section

*Synthesis of Hierarchically Mesoporous TiO_2_ Nanowire Bundles*: This process was very simple and could easily be scaled up to large‐scale production. In a typical synthesis of the hierarchically mesoporous TiO_2_ nanowire bundles (HM‐TiO_2_‐NB), 0.5 g of P123 and 1 mL of concentrated HCl were added to 20 mL of anhydrous ethanol under vigorous stirring. Then, 2.96 mL of TTIP was added to the above solution. The final mixture was further stirred in a 50 mL beaker for 12 h at room temperature. The beaker was then placed in an oven at 40 °C with humidity over 60 RH% for 12 h and subsequently heated at 80 °C for 12 h. Finally, the product was washed using ethanol reflux method for 12 h and dried at 60 °C.

*Characterizations*: XRD patterns were obtained on a Bruker D8 system with Cu Κα radiation (*λ* = 0.15405 nm) with 40 mA and 40 kV. SEM observation was carried out using an S‐4800 field‐emission SEM (FESEM, Hitachi, Japan). TEM and HRTEM images and SAED patterns of the samples were recorded on carbon‐coated copper grids by using a JEM‐2100F microscope at an acceleration voltage of 200 kV (JEOL, Japan). Nitrogen adsorption–desorption isotherms were obtained using a TriStar II 3020 surface area and porosity analyzer at 77 K (Micromeritics, USA). The specific surface area was calculated by the Brunauer–Emmett–Teller (BET) method. The pore size distribution was calculated by the Barret–Joyner–Halenda (BJH) method. Raman measurements were carried out at room temperature, and the signals were recorded by an Invia Raman Microscope (Invia Microscope, Renishaw, UK) using the 632.8 nm line of a Nd YAG laser as the excitation source. The surface electronic states of the Ti element were analyzed by XPS (VG Multilab 2000). Tap density was measured by the BT‐301 tap denser. The as‐prepared powders were poured into 25 mL glass cylinder, and this cylinder was tapped for 10 min with 300 taps min^−1^ frequency.

*Electrochemical Characterization*: Electrochemical experiments were performed with Swagelok‐type cells with pure lithium metal as both the counter electrode and the reference electrode at room temperature. The working electrode consisted of active material (HM‐TiO_2_‐NB), a conductive agent (carbon black, super‐P), and a polymer binder (poly vinylidenedifluoride) (PVDF) in a 70:20:10 weight ratio. After these materials had been thoroughly mixed in an *N*‐methyl‐2‐pyrrolidone (NMP) solution, the prepared slurry was coated on Cu foil. The coated electrode was dried at 120 °C in a vacuum oven for 12 h. Circular disk electrodes were punched from the foil and a lithium metal foil was used as the counter electrode. The electrolyte used was 1.0 m LiPF6 in a 50:50 (w/w) mixture of ethylene carbonate and diethyl carbonate. Cell assembly was carried out in an argon‐filled glove box. Cyclic voltammetry (1–3 V) was performed using an electrochemical workstation (CHI 660C). The charge–discharge tests were performed using a battery tester (LAND) with a voltage window of 1–3 V at various current rates.

## Supporting information

As a service to our authors and readers, this journal provides supporting information supplied by the authors. Such materials are peer reviewed and may be re‐organized for online delivery, but are not copy‐edited or typeset. Technical support issues arising from supporting information (other than missing files) should be addressed to the authors.

Supplementary
