# Supplementary material for: Phases Hybriding and Hierarchical Structuring of Mesoporous TiO2 Nanowire Bundles for High‐Rate and High‐Capacity Lithium Batteries
Source: Adv Sci (Weinh). 2015 May 8;2(7):1500070. doi: 10.1002/advs.201500070 (PMC5029796; doi:10.1002/advs.201500070)
Supplement: Supplementary file 1 — Supplementary [file ADVS-2-0h-s001.pdf]

Copyright WILEY-VCH Verlag GmbH & Co. KGaA, 69469 Weinheim, Germany, 2015.

ADVANCED SCIENCE
Open Access

Supporting Information

for *Adv. Sci.*, DOI: 10.1002/advs.201500070

Phases Hybriding and Hierarchical Structuring of Mesoporous TiO_2 Nanowire Bundles for High-Rate and High-Capacity Lithium Batteries

Jun Jin, Shao-Zhuan Huang, Jing Liu, Yu Li, Li-Hua Chen, Yong Yu, Hong-En Wang, Clare P. Grey, and Bao-Lian Su**

Supporting Information

Phases Hybriding and Hierarchical Structuring of Mesoporous TiO_2 Nanowire Bundles for High Rate and High Capacity Lithium Batteries

Jun Jin[a], Shao-Zhuan Huang[a], Jing Liu [a], Yu Li[a], Li-Hua Chen[a], Yong Yu[a], Hong-En Wang[a] Clare P. Grey[c] and Bao-Lian Su*[a,b,c]*

Prof. Y. Li, Prof. B. L. Su, J. Jin, S. Z. Huang, J. Liu, Prof. L. H. Chen, Dr. Y. Yu, Prof. H. E. Wang

[a]Laboratory of Living Materials at the State Key Laboratory of Advanced Technology for Materials Synthesis and Processing, Wuhan University of Technology, 122 Luoshi Road, 430070, Wuhan, Hubei, China; Fax:+86 27 87879468; Tel: +86 27 87855322

Email: yu.li@whut.edu.cn; baoliansu@whut.edu.cn

Prof. B. L. Su

[b]Laboratory of Inorganic Materials Chemistry (CMI), University of Namur, 61 rue de Bruxelles, B-5000 Namur, Belgium; Fax: +32 81 725414; Tel: +32 81 724531

E-mail: bao-lian.su@unamur.be

Prof. B. L. Su and Prof. Clare P. Grey

[c]Department of Chemistry, University of Cambridge

E-mail: bls26@cam.ac.uk; cpg27@cam.ac.uk

Supplementary Figures and Captions

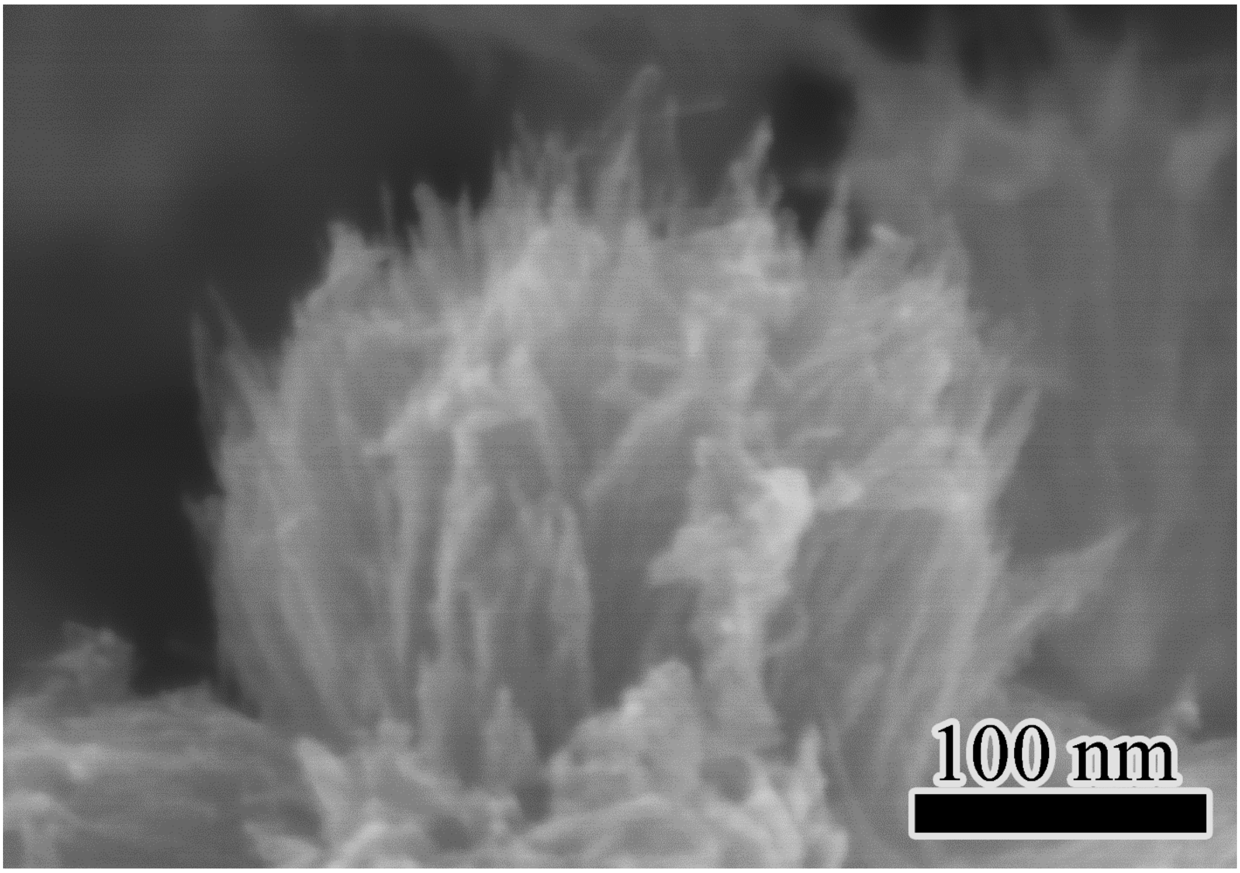

Figure S1. The high resolution SEM image of the as-prepared HM-TiO_2-NB sample, clearly showing the petal-like structures of the HM-TiO_2-NB material consisted of plenty of small one-dimensional (1D) nanowires.

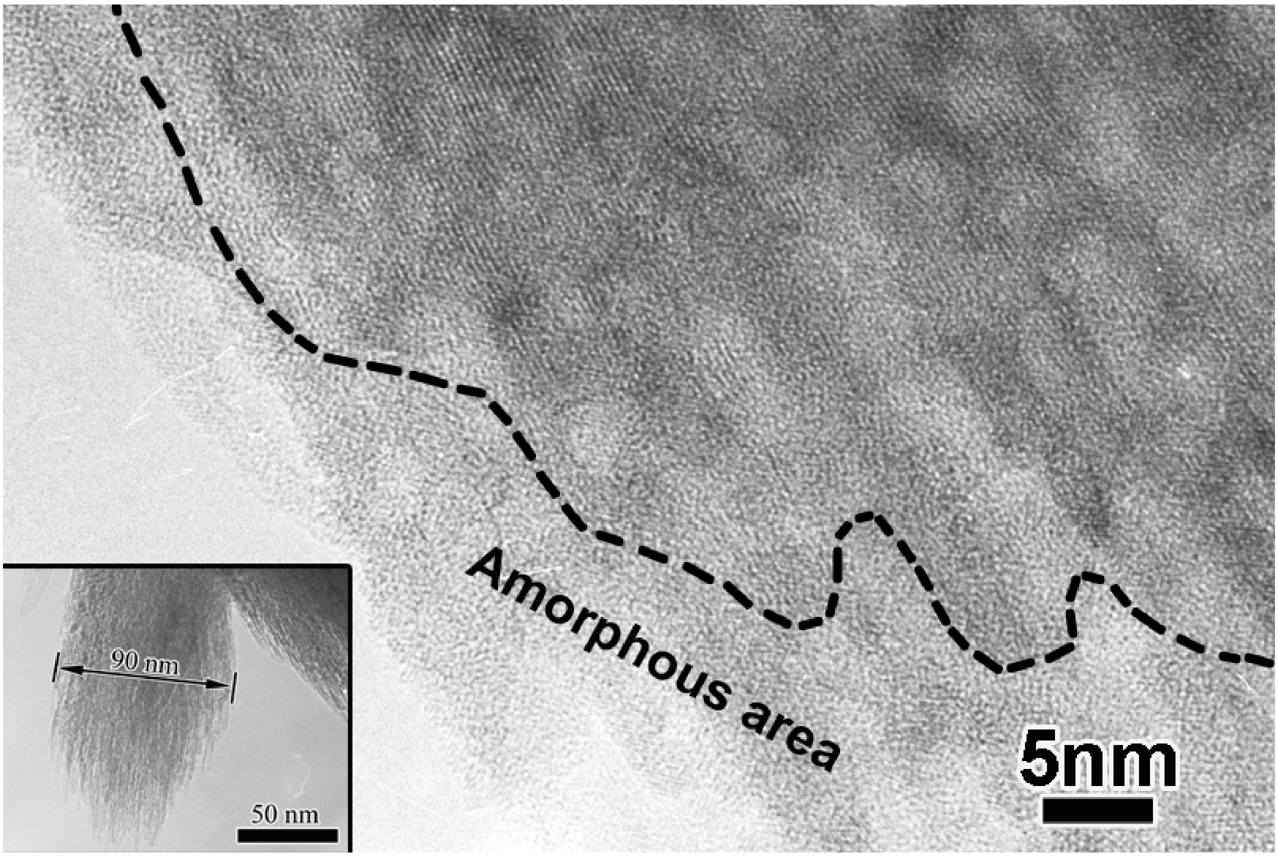

Figure S2. The close view at the tip of the bundle shown in Figure 1f, clearly showing the amorphous layer in each nanowire.

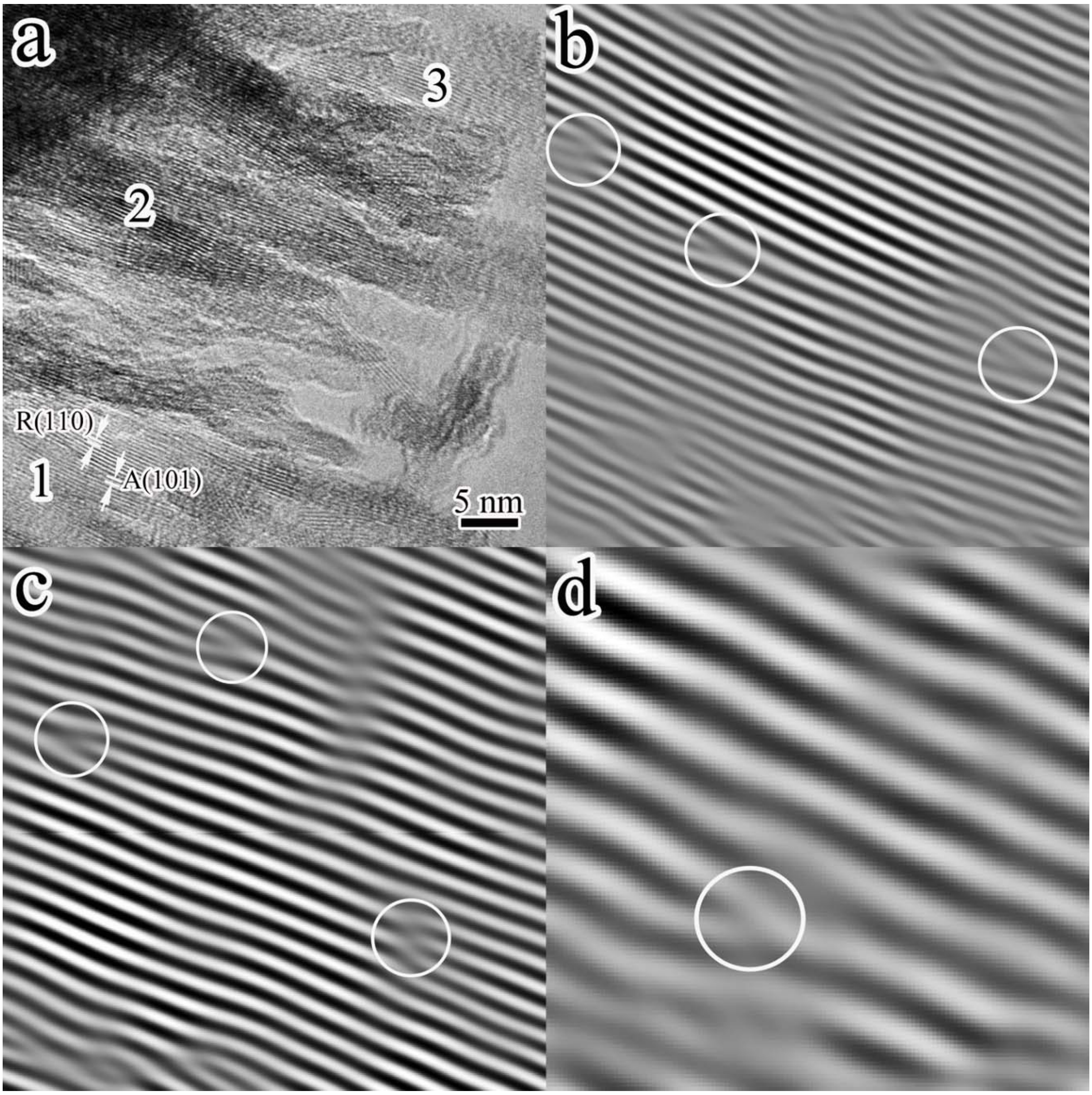

Figure S3. The HRTEM image and the corresponding FFT transformation images of the as-prepared HM-TiO_2-NB sample. (a) the rutile and anatase phases appearance in the same nanowire; (b) FFT transformation image of the edge dislocations in area 1; (c) FFT transformation image of the edge dislocations in area 2; (d) FFT transformation image of the edge dislocations in area 3. The FFT transformation images clearly show the amorphous TiO_2 on the surface.

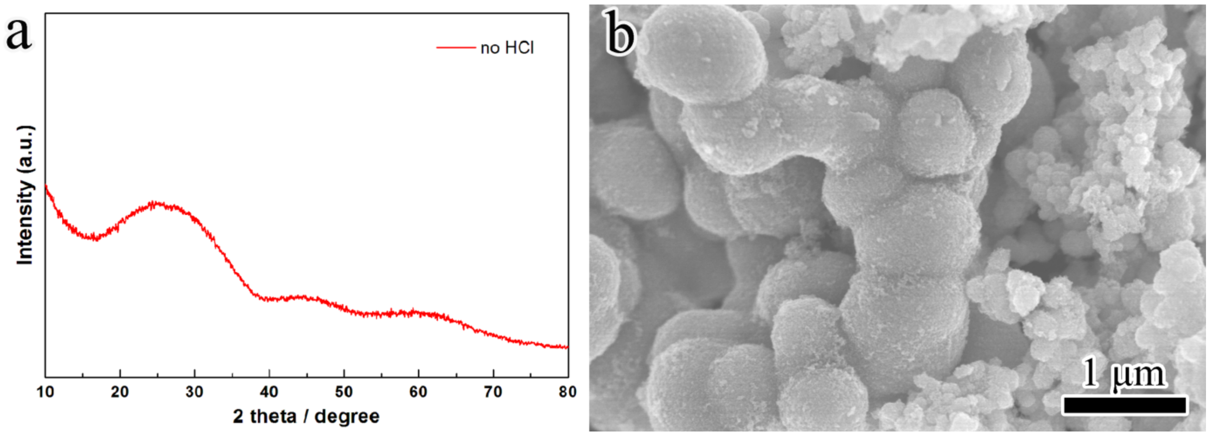

Figure S4. (a) XRD pattern, (b) SEM image of the prepared TiO_2 sample without hydrochloric acid added in the reaction system.

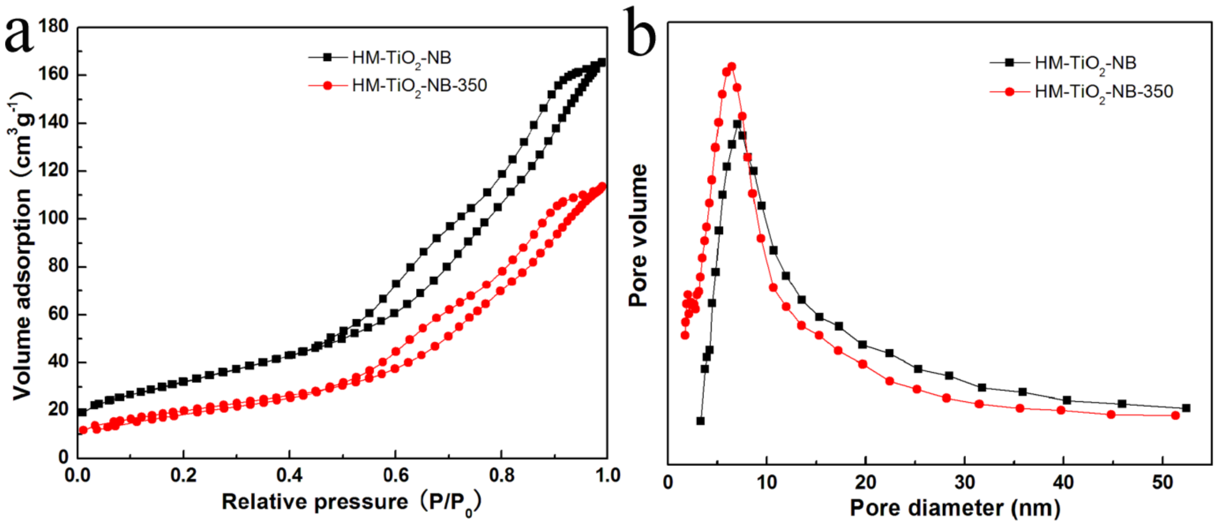

Figure S5. (a) Nitrogen adsorption / desorption isotherms and (b) the pore size distribution plots of the as-prepared HM-TiO_2-NB and HM-TiO_2-NB-350 samples.

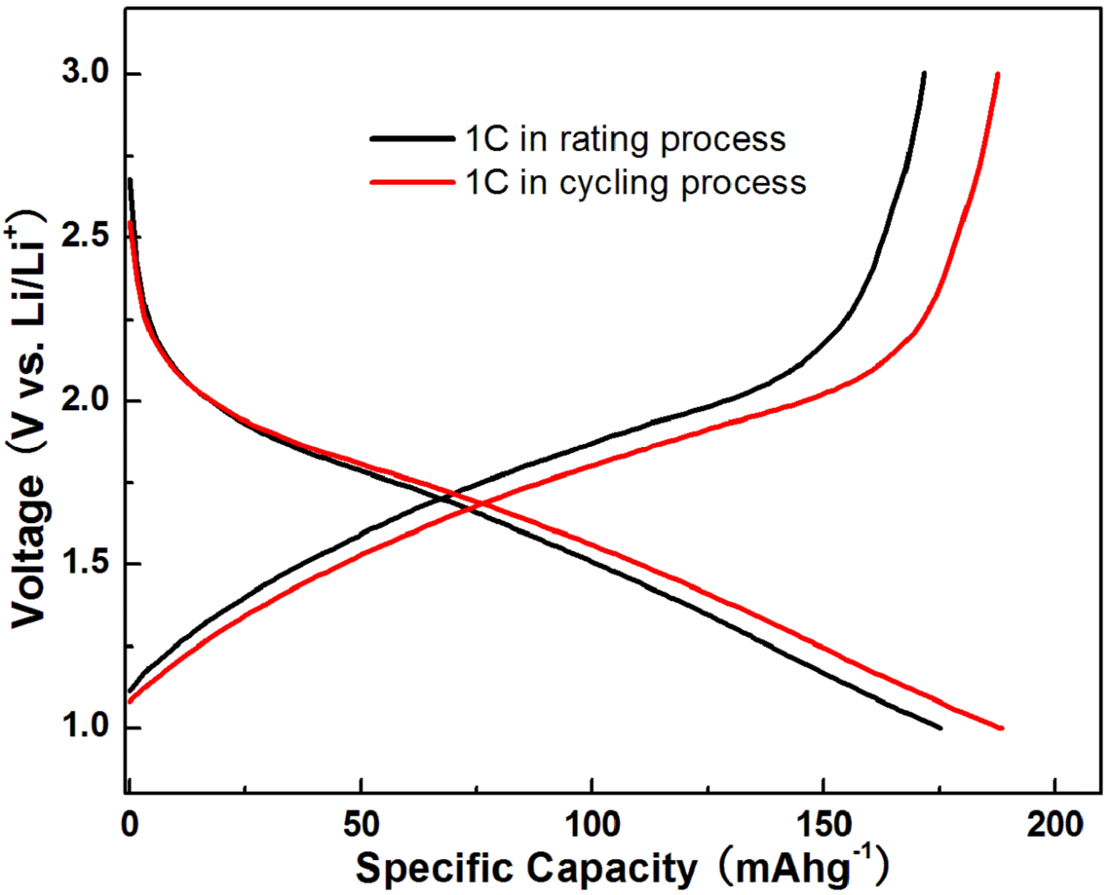

Figure S6. Discharge-charge profiles of anode material at 1 C in the rating and cycling processes.

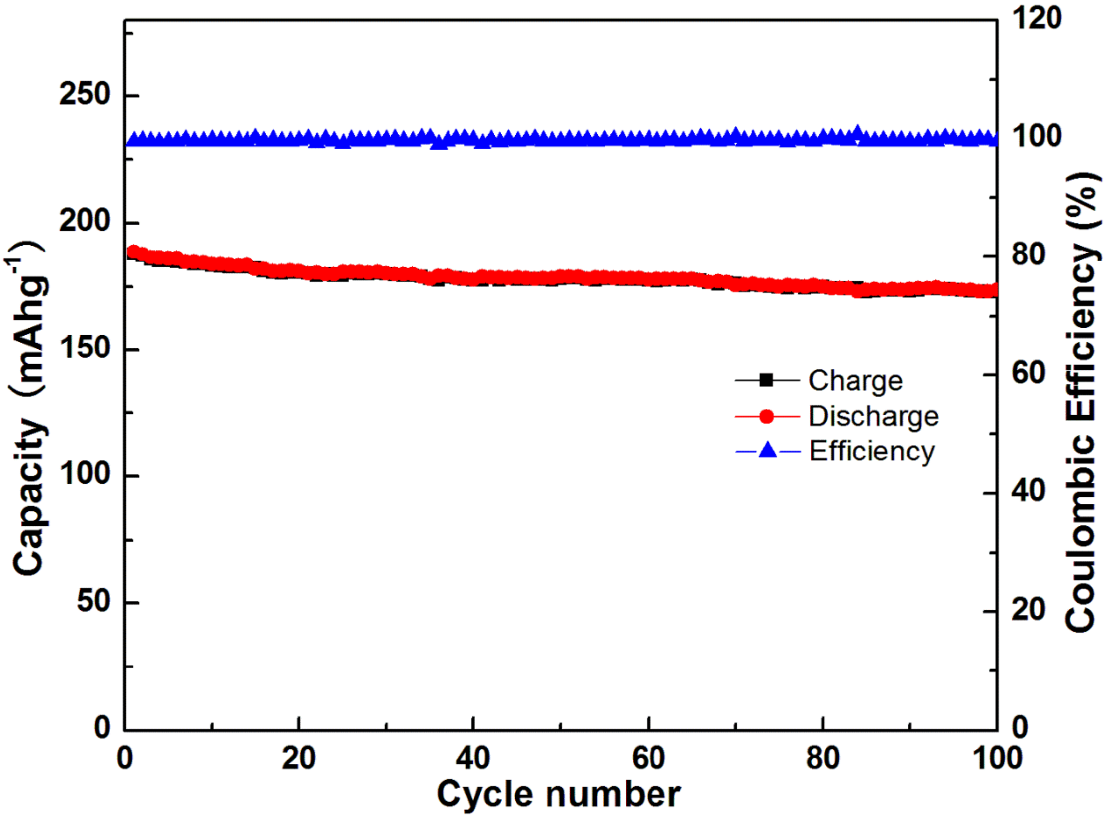

Figure S7. Cycling performance and coulombic efficiency at 1 C for 100 cycles.

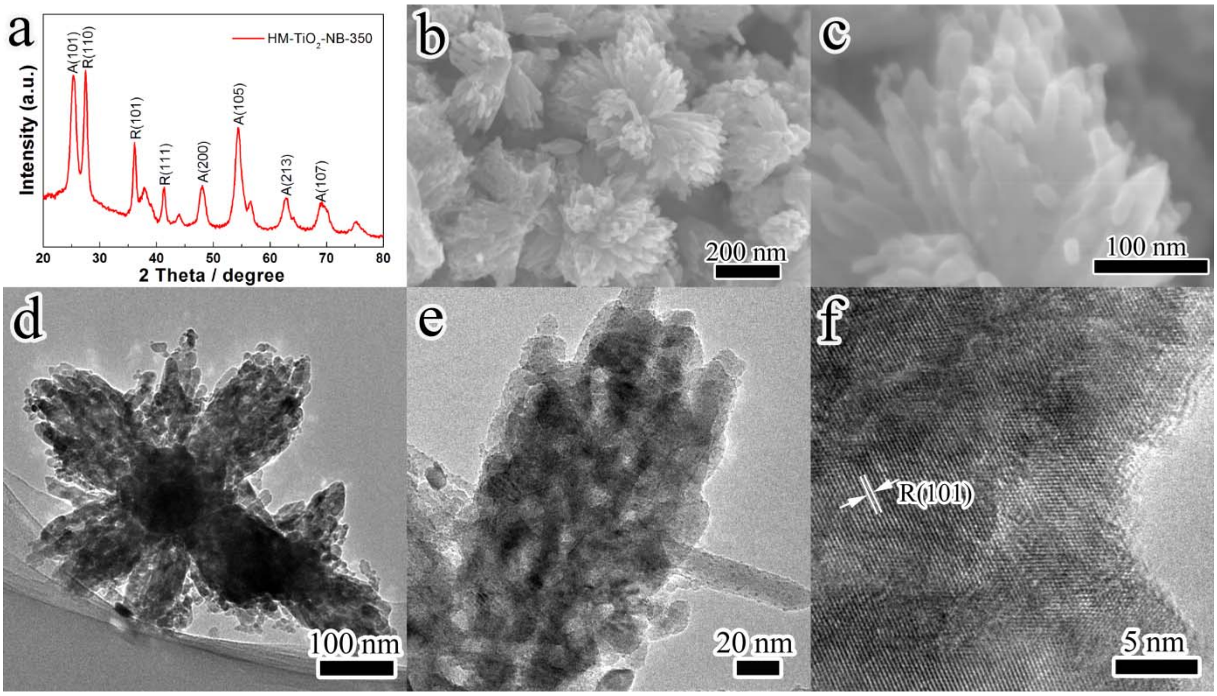

Figure S8. The characterizations of the HM-TiO_2-NB-350 sample. (a) XRD patterns; (b) and (c) SEM images; (d) and (e) TEM image; (f) HRTEM images.
